# Local adaptation shapes functional traits and resource allocation in black spruce

**DOI:** 10.1038/s41598-023-48530-6

**Published:** 2023-12-01

**Authors:** R. Silvestro, C. Mura, D. Alano Bonacini, G. de Lafontaine, P. Faubert, M. Mencuccini, S. Rossi

**Affiliations:** 1 Laboratoire sur les écosystèmes terrestres boréaux, Département des Sciences Fondamentales, Université du Québec à Chicoutimi, 555 Boulevard de l’Université, Chicoutimi, QC G7H2B1 Canada; 2 Canada Research Chair in Integrative Biology of the Northern Flora, Département de biologie, chimie et Géographie, Centre for Northern Studies, Centre for Forest Research, Université du Québec à Rimouski, Rimouski, QC Canada; 3 Carbone boréal, Département des Sciences Fondamentales, Université du Québec à Chicoutimi, 555 Boulevard de l’Université, Chicoutimi, QC G7H 2B1 Canada; 4 Centre de Recerca Ecològica i Aplicacions Forestals (CREAF), 08193 Bellaterra, Barcelona, Spain; 5 Institució Catalana de Recerca i Estudis Avançats (ICREA), Passeig de Lluis Companys 23, 08010 Barcelona, Spain

**Keywords:** Forest ecology, Boreal ecology

## Abstract

Climate change is rapidly altering weather patterns, resulting in shifts in climatic zones. The survival of trees in specific locations depends on their functional traits. Local populations exhibit trait adaptations that ensure their survival and accomplishment of growth and reproduction processes during the growing season. Studying these traits offers valuable insights into species responses to present and future environmental conditions, aiding the implementation of measures to ensure forest resilience and productivity. This study investigates the variability in functional traits among five black spruce (*Picea mariana* (Mill.) B.S.P.) provenances originating from a latitudinal gradient along the boreal forest, and planted in a common garden in Quebec, Canada. We examined differences in bud phenology, growth performance, lifetime first reproduction, and the impact of a late-frost event on tree growth and phenological adjustments. The findings revealed that trees from northern sites exhibit earlier budbreak, lower growth increments, and reach reproductive maturity earlier than those from southern sites. Late-frost damage affected growth performance, but no phenological adjustment was observed in the successive year. Local adaptation in the functional traits may lead to maladaptation of black spruce under future climate conditions or serve as a potent evolutionary force promoting rapid adaptation under changing environmental conditions.

## Introduction

Climate change affects the weather by raising temperatures, shifting precipitation regimes, and producing more frequent and intense extreme events^1–3^. These changes are expected to shift the climatic zones^4^ at a magnitude that exceeds the natural rate of vegetation migration^5^. Such a shift of climatic niches challenges one of the fundamental assumptions underlying past and present forest management, which affirms that native seed sources must be preferred to those from other regions because they have a better adaptation to the local conditions^6^. The ongoing geographical shifts in climate can mismatch the trees from their optimal niche, causing maladaptation^7^. For this reason, the current challenge for forest managers is predicting climate change impacts on the vegetation and implementing measures to sustain resilience, functionality and productivity of forest ecosystems. However, such an aim requires a deep understanding of the complex relationships between climate and the eco-physiological processes of trees^8^, which are still partially lacking for many species.

The survival of trees in a specific location relies on functional traits, which encompass the morphological, physiological and phenological attributes governing their interactions and responses to the environment^8–10^. Within certain species, local populations exhibit divergent phenology, as well as growth and reproductive performances that are adapted to optimize survival and reproductive success in their specific local conditions^11^. The intraspecific variation of functional traits reflects differences in genotype and phenotypic plasticity across environments^12^. Local adaptation to contrasting environments driven by natural selection leads to an increased genetic divergence over time^12^. Plasticity enables individuals to quickly adjust their phenotypes in response to local conditions, thereby mitigating selective pressures^13^. Genetic variation and plasticity also play crucial roles in determining the ability of populations to adapt to climate change^14^, including the opportunity of moving genotypes or provenances for restoration and reforestation practices.

In recent decades, scientists have focussed on the intraspecific variability of phenological and growth traits^15^. Phenological traits determine the seasonal timings of biological events, ensuring growth and reproduction, while minimizing the risks due to climate hazards^16,17^. Tree size, particularly total height at a specific age, is commonly used in forestry as an indicator of fitness and productivity^18^. These traits can offer valuable insights into species responses to current and future environmental conditions. In addition, adaptations in phenology and growth performance can reflect intraspecific differences in life history and resource allocation strategies, potentially encompassing other key functional traits. However, while it is important to recognize that some traits could be correlated with each other, the sensitivity and responsiveness to climate factors could vary between co-evolved traits^10,19^. Therefore, we stress the importance of assessing multiple traits when quantifying biological and ecological variations within a species.

Compared to other functional traits, very little is known about the response of plant reproduction to climate change^20^. There is a lack in the literature on lifetime first reproduction, although such a trait is closely related to plant demography^21^. The generation time is a demographic parameter that can influence the rate at which species or populations evolve. The earlier the beginning of reproduction, the higher the ability of populations to respond to a changing environment^22^. Lifetime first reproduction is intimately linked to growth performances. Indeed, it is widely acknowledged that trees need to reach a certain age or size to produce flowers and seeds^23^. As different provenances can show different growth performances^24–26^, we raise the hypothesis that lifetime first reproduction, or the size to initiate cone production, changes with provenance.

Frost hardiness is an important trait influenced by phenology and affecting fitness and growth performance^27–29^. Frost hardiness changes during the year, being at a minimum during the growing season and reaching its maximum during dormancy, when temperatures are lower^30^. Budbreak is a transition between dormancy and growth, and needs to be synchronized with environmental conditions in order to avoid the risk of frost damage on vulnerable new tissues^30^. Under climate change, warmer spring temperatures cause an advance in spring phenology, increasing the risk of exposing the young shoots to damaging late-spring frost events^30,31^. Late-spring frost damage can occur when below-freezing temperatures hit after budbreak or the first leaf-out. Exposure to frost during the initial stages of leaf emergence can have dramatic effects on growth and reproduction by affecting individual resource acquisition^28,32,33^. Plants experiencing a non-lethal stress can respond with a phenological shift in the subsequent year^34–36^. This shift typically involves a sugar-mediated response, manifested by earlier starch breakdown^35^. The higher sugar availability for shoot growth explains the phenological shift of budburst in defoliated individuals. Since frost damage primarily affects crown development, a similar phenological shift may occur in individuals with a significant degree of damaged buds, potentially increasing or decreasing frost hardiness.

This study relies on a common garden experiment to investigate the variation in adaptive traits of five black spruce [*Picea mariana* (Mill.) B.S.P.] provenances originating from a latitudinal gradient in the coniferous boreal forest in Quebec, Canada. Specifically, we assess the differences among provenances in: (1) bud phenology and growth performance, (2) timings of lifetime first reproduction, and (3) the consequences of the late-frost event in 2021 on the growth performance and phenological adjustments of trees. According to previous studies^24,37^, the provenance differs in bud phenology and growth performance. We test the common assumption that the provenances with faster growth start reproduction at younger ages^38^. Lastly, we expect that the trees experiencing damage from frost show an annual growth decline but adjust their phenology to mitigate exposure to late frost events during the successive years.

## Results

**Figure 1:**
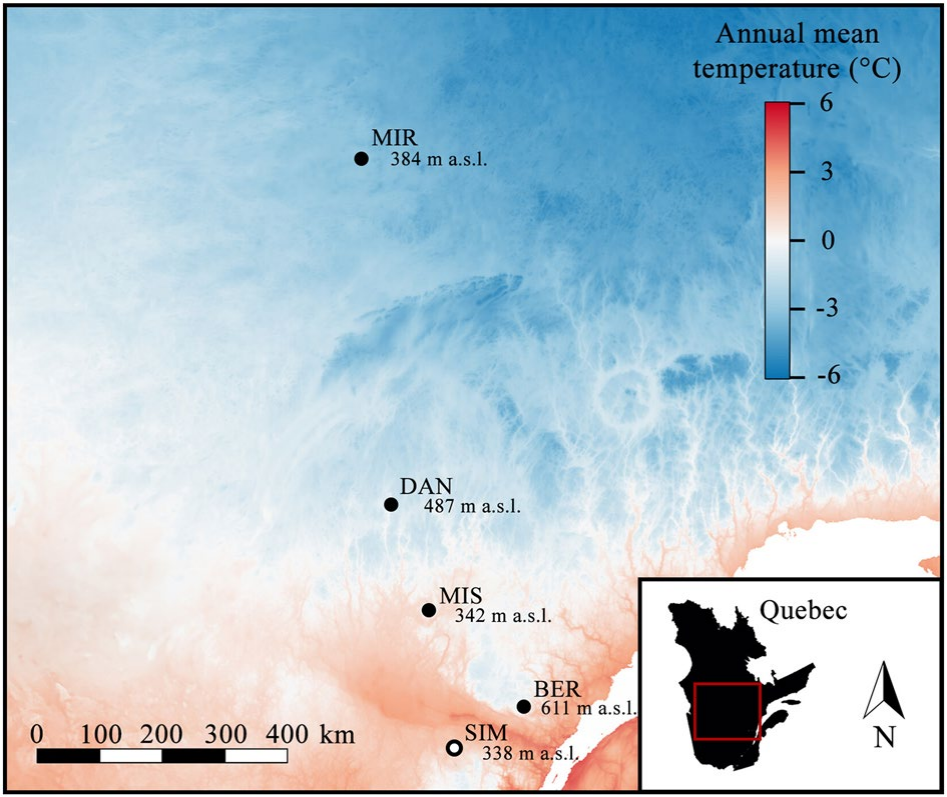
Origin sites of the five black spruce provenances [Simoncouche (abbreviated as SIM), Bernatchez (BER), Mistassibi (MIS), Camp Daniel (DAN) and Mirage (MIR)] in Quebec, Canada, and annual temperature across the study region. All provenances were planted in a common garden located in SIM (white circle with black border).

In this study, we assessed the timings of bud development, apical growth, reproductive maturity, and the damage caused by late-spring frost in five provenances of black spruce in a common garden experiment. These provenances originated from natural stands located along a latitudinal gradient ranging from the 48th to 53rd parallels in Quebec (Fig. 1).

### Phenological timings and growth performances

Bud break occurred from the beginning of May to mid-June, while bud set from the end of June to the end of September, depending on the study year and provenance (Fig. S1; Table S1). On average, the period of bud break lasted 36 days, with a difference of 16 days between the shortest (28 days) and longest (44 days) period in 2020 and 2022, respectively (Fig. S2). The duration of bud set was more variable among years. On average, bud set lasted 74 days, with a difference of 42 days between the shortest (53 days) and longest (95 days) period in 2019 and 2021, respectively (Fig. S2). The phenological stages were delayed by 1.5–2.1 days for each degree Celsius of increase in mean annual temperature of the origin site.

The duration of growth (i.e., of the period required for apical shoot extension) occurred from the beginning of June to the beginning of July and was longer in the provenances originating from the coldest sites (Fig. S2). On average, the period of shoot extension was shorter by 0.18–1.77 days for each degree Celsius of increase in mean annual temperature of the original site.

**Figure 2:**
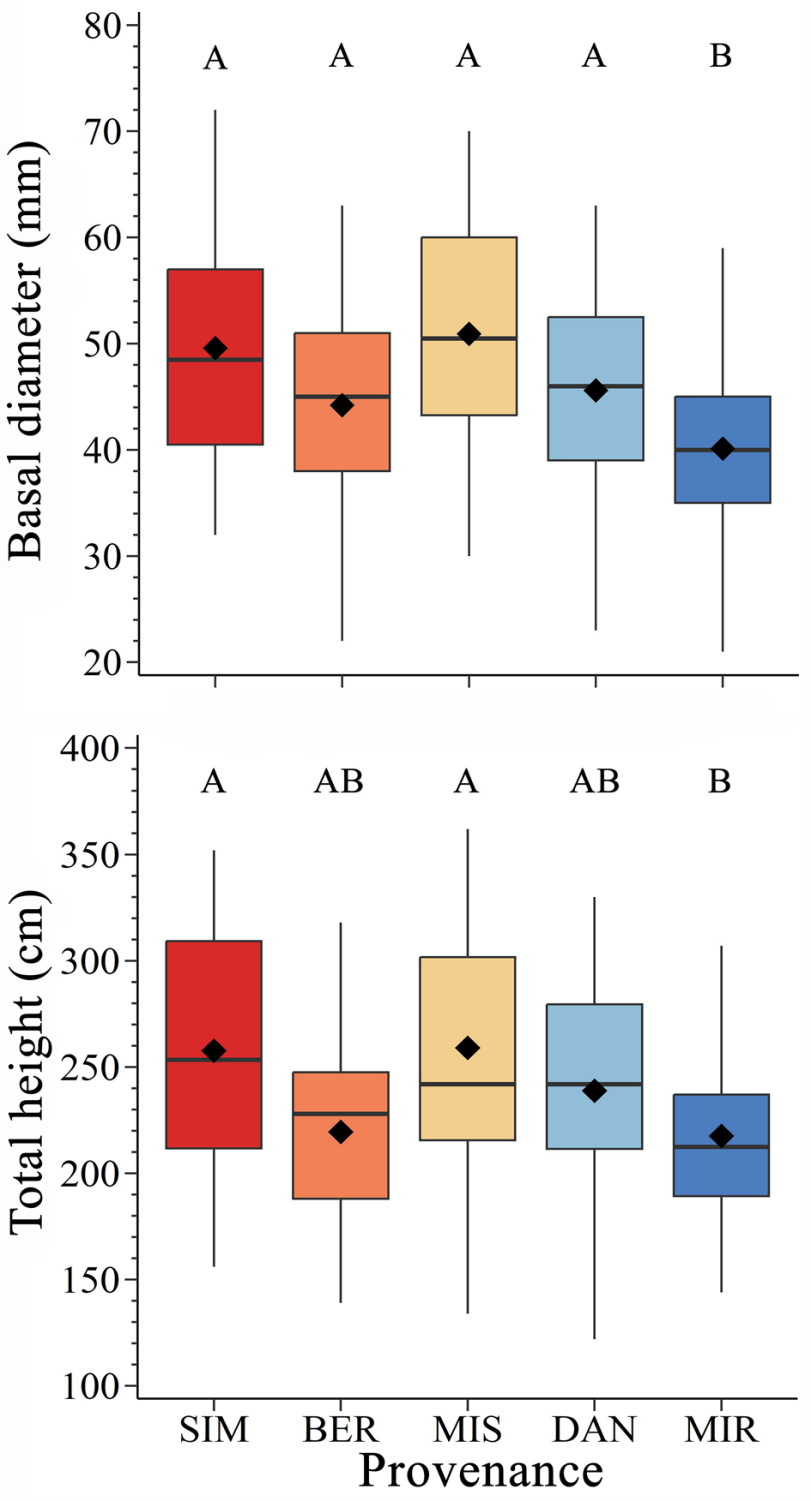
Comparison of basal diameter and total height across the five black spruce provenances [Simoncouche (abbreviated as SIM), Bernatchez (BER), Mistassibi (MIS), Camp Daniel (DAN) and Mirage (MIR)]. Boxplots represent upper and lower quartiles; whiskers achieve the 10th and 90th percentiles; the median and mean values are drawn as horizontal black lines and black diamond, respectively.

The annual height increment varied according to provenance, with provenances from the northern sites exhibiting lower shoot extension compared to provenances from the southern sites (Fig. S3). The effect of provenance was also confirmed on the total height in 2022 (*F* = 4.83, *P* < 0.001). On average, individuals from the northernmost provenance (MIR) were 40 cm shorter than those from the southernmost provenance (SIM), with an average height of 212 and 252 cm, respectively (Fig. 2).

The effect of provenance was also confirmed on the basal diameter in 2022 (*F* = 9.13, *P* < 0.001). On average, the basal diameter of individuals from MIR was 10 mm smaller than those from SIM, with an average basal diameter of 40 and 50 mm, respectively (Fig. 2).

### Lifetime first reproduction

**Figure 3:**
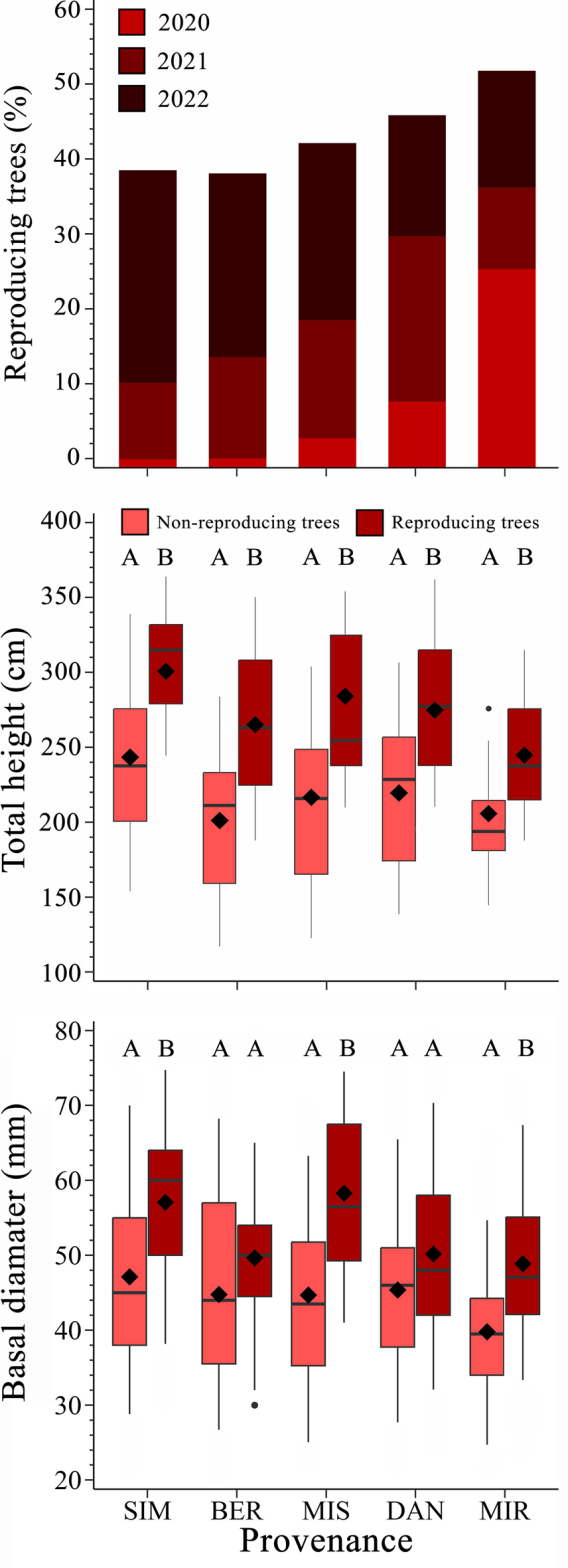
Number of trees in reproduction and tree growth in the five black spruce provenances [Simoncouche (abbreviated as SIM), Bernatchez (BER), Mistassibi (MIS), Camp Daniel (DAN) and Mirage (MIR)]. The boxplots illustrate the upper and lower quartiles, with whiskers indicating the 10th and 90th percentiles. The median and the mean are represented by horizontal black lines and a black diamond, respectively.

Provenances from the northern sites reached reproductive maturity earlier than those from the southern sites (Fig. 3). In 2020, the first year in which cones were observed in the studied trees, 26% of individuals from the northernmost provenance (MIR) had cones on the crown. By 2022, 52% of trees from MIR showed cones, while only 38% of trees from the southernmost provenance SIM had started reproduction (Fig. 3). We also found that reproducing trees were taller (*F* = 56.07, *P* = 0.001) and had a larger basal diameter (*F* = 35.70, *P* = 0.001) (Fig. 3, Table S2). On average, reproductive trees were 55 cm taller and 9 mm larger in diameter than non-reproducing trees from the same provenance (Fig. 3).

### Late frost event and frost damages

The late frost event took place on 28 and 29 May, 2021 (DOY 148–149), when nighttime temperatures at the common garden in SIM dropped sharply, reaching − 1.1 and − 1.9 °C, respectively. The percentage of damaged trees followed the temperature gradient, ranging from 60.7 to 100% for the southernmost (i.e., SIM) and northernmost (i.e., MIR) sites, respectively (χ^2^ = 58.98, *P* < 0.0001). Overall, low levels of damage (i.e., < 15% of damaged buds) were more common, accounting for 75.7% of all observations. Only 15.6% of trees showed no damage, 79.3% of which belonged to the two southernmost provenances (i.e., SIM and MIS). Provenances from the northernmost sites exhibited higher levels of damage (i.e., > 15% of damaged buds), with the two northernmost provenances (i.e., MIR and DAN) accounting for 71.8% of all trees with highly damaged trees.

### Frost damage and bud phenology

Compared to the other study years, the onset and ending of budbreak occurred earlier in 2021, while development of the winter bud took longer, with an earlier onset and delayed ending of bud set (Fig. S1, S2). In 2021, the onset of budbreak in the two northernmost provenances (i.e., MIR and DAN) occurred on average on DOY 135, 13 days before the late frost event.

**Figure 4:**
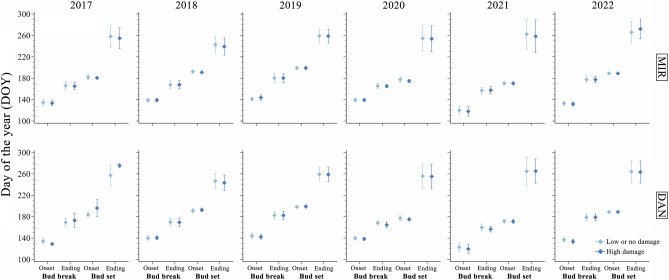
Bud phenology in trees according to their visible damage by the late frost in 2021. Mean values are denoted by dots, while vertical lines indicate the standard deviations.

For the two provenances most damaged by the frost (i.e., MIR and DAN), the timings of budbreak and bud set varied between years (Fig. 4, Table S3), with significant differences observed for all phases, (*F*-values ranging between 44.67 and 352.58, *P* < 0.001) (Table S3). However, no effect of the damage level or the interaction between damage level and year was observed (Table S3). Therefore, we found no significant differences in phenology between damaged and undamaged trees (Fig. 4).

### Frost damage and height growth performances

**Figure 5:**
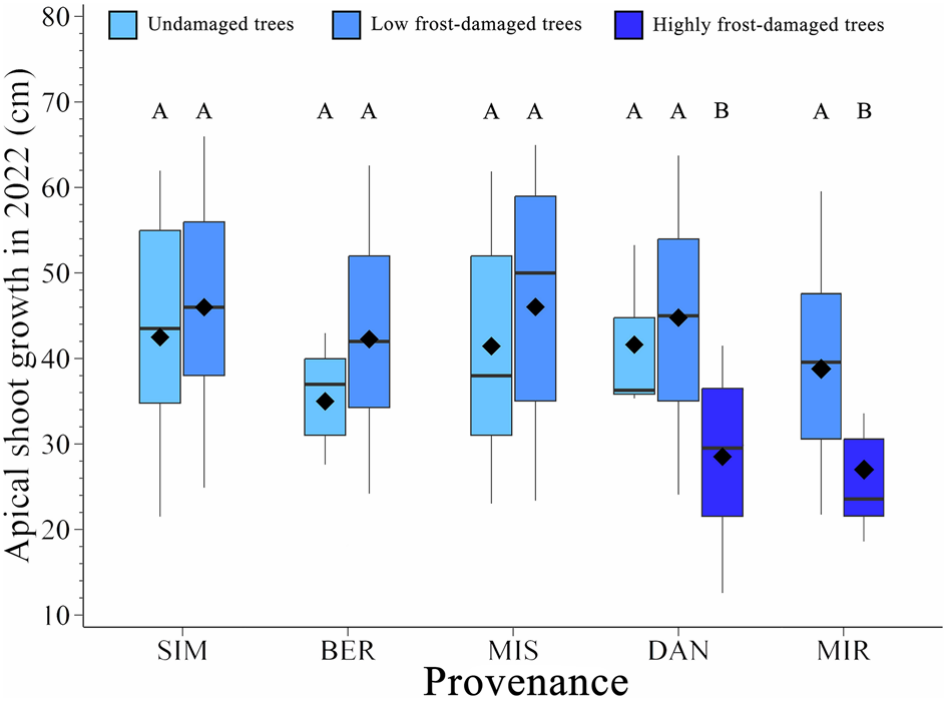
Apical shoot extension in 2022 in trees with different damage by the late frost in 2021. The boxplots illustrate the upper and lower quartiles, with whiskers indicating the 10th and 90th percentiles. The median and the mean are represented by horizontal black lines and a black diamond, respectively.

The effects of provenance, damage level, and their interaction on apical shoot growth were significant (*P* < 0.01) (Table S4). In particular, provenances with a higher level of damage (i.e., > 15% of damaged buds) exhibited a lower apical shoot growth (26 cm) compared with provenances with low or no damage (42 cm) (Fig. 5).

**Figure 6:**
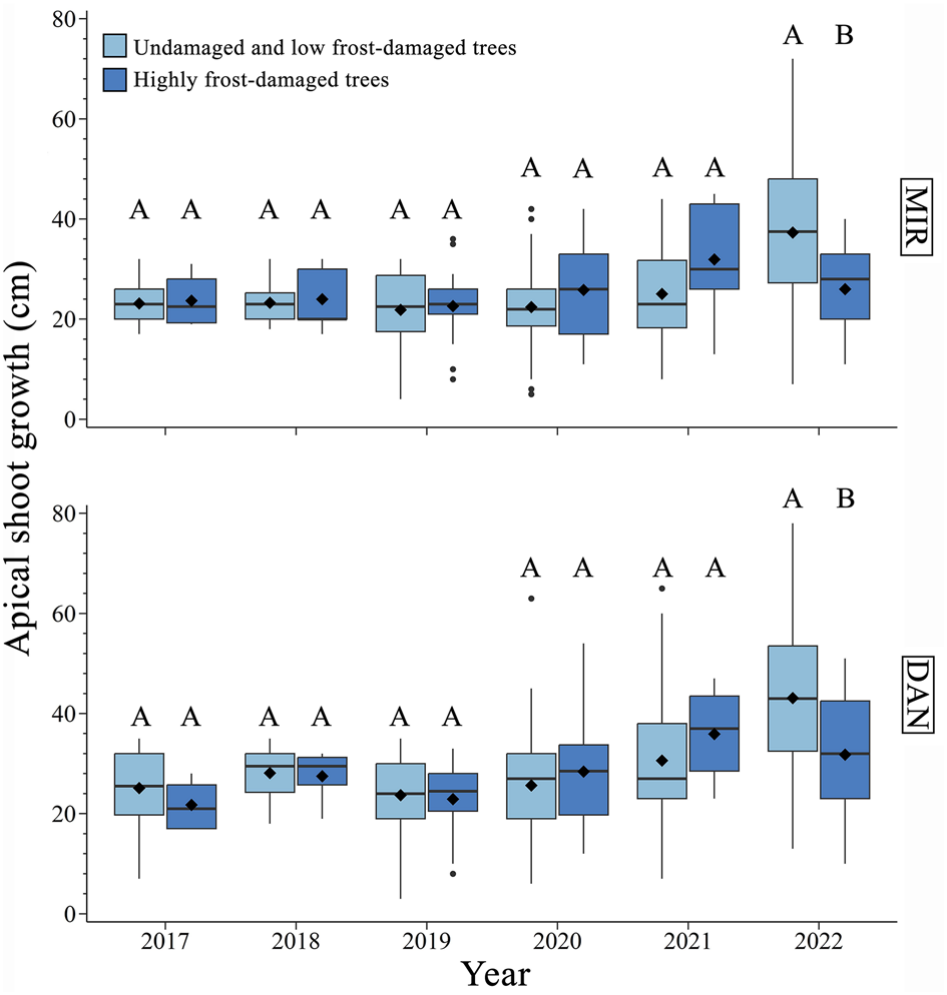
Apical shoot extension in trees with different damage by the late frost in 2021 for the study years 2017–2022. The boxplots illustrate the upper and lower quartiles, with whiskers indicating the 10th and 90th percentiles. The median and the mean are represented by horizontal black lines and a black diamond, respectively.

The growth in height in the two provenances most damaged (MIR and DAN), varied between years (Fig. 6, Table S5). This difference was significant (*F*-value in MIR = 18.99, *F-*value in DAN = 28.32, *P* < 0.001 for both provenances) (Table S5). However, the growth between highly damaged trees and those with low or no damage was similar across all study years, except for 2022, when a significant difference was observed (Fig. 6).

## Discussion

This study relies on a common garden experiment to test differences in functional traits among five black spruce provenances originating from a latitudinal gradient along the boreal forest of Quebec, Canada. We profit from the late frost event occurring in 2021 to assess differences in frost tolerance and investigate the successive growth performance and phenological adjustment in trees. The results reveal significant variations in all observed traits among the provenances. Trees from the northern sites have earlier bud development, lower growth increments, and reached reproductive maturity earlier than those from southern sites. The late frost in 2021 affected growth performance, but no phenological adjustment was observed.

### Tree size and reproductive maturity

Northern provenances exhibited an earlier reproductive maturity than those from southern regions. Trees need to reach a specific age or size before initiating cone production, which aligns with existing knowledge^23^, although cone production does not automatically imply that the seeds are viable and fertile. However, our findings reveal that the size for reproductive maturity varies among provenances. Northern provenances attain reproductive maturity at smaller dimensions than those originating from the southern sites. In particular, northern provenances exhibit cones at a smaller height than southern provenances.

In trees, the size for reproduction reflects the necessity to allocate the resources to growth during the early developmental stages to successively maximize reproductive success^39^. For certain coniferous species, tree size has proven to be a better indicator of cone production than tree age^40,41^. This relationship is likely explained by the fact that tree size strongly reflects the access to resources^42^. Consequently, within a provenance, the correlation between total height and reproductive maturity might be attributed to intra-provenance variability in growth performance and individual fitness^43^.

Among provenances, the variability in the threshold size to reach reproductive maturity probably reflects a divergent life history strategy and resource allocation. Generally, the risk of mortality and its predictability influence the timing of first reproduction within species^44,45^. For example, an early high fecundity in fire-prone ecosystems is an advantageous trait^46^. In such ecosystems, the persistence of a species after recurrent wildfires (i.e., stand self-replacement) is usually contingent on immediate and vigorous post-fire tree recruitment^46,47^. Post-fire seeding species, such as black spruce, rely on the ability to quickly establish a persistent seed bank before the subsequent wildfire event^48^. In this context, the higher wildfire frequency at higher latitudes in Eastern Canada^49^ likely represents a selective force advancing the reproductive maturity of northern provenances, as highlighted in our study.

Differentiation between provenances in reproductive allocation and growth performances can therefore be regarded as a fingerprint of different selection processes acting in their respective native environment. It is well known that, when moved south, growth performance of northern provenances remains lower than that of southern ones^18,24^. In this context, the variability in certain functional traits within a species may reflect some more conservative and precautionary strategies, whereby northern provenances invest more resources during their life cycle to ensure individual survival (e.g., maintaining higher levels of frost hardiness during the dormant season) and enhance reproductive success.

### Phenology and frost tolerance

Late frost affected the growth performance but did not trigger a phenological adjustment. As emphasized in a previous study, northern provenances were more exposed to the late frost due to their earlier growth reactivation and budbreak^50^. Accordingly, the extent of damage in northern provenances exceeded that observed in provenances from the southern sites^50^. Our findings highlight the existence of a threshold level of damage that results in a decline in height growth. In particular, individuals from the two northern provenances (i.e., MIR and DAN), which had a higher proportion of buds damaged, experienced the larger contraction in height growth in the following year.

The impact of a frost on growth performance has primarily been studied in terms of radial growth. Late frosts occur at the onset of the growing season, and often lead to reductions in tree ring width comparable to, or greater than, those caused by extreme summer droughts^51,52^. However, no carry-over effects are reported in the subsequent years^53^. In this study, we observe that the effects of the late frost on height growth appears only in the subsequent growing season. There are several explanations for this delayed impact on height growth. Previous research has demonstrated that the canopy recovers in 2 months from a late frost event^54,55^, suggesting a potential recovery through exact compensation^56^. Still, canopy recovery comes at a cost in terms of resource allocation. Several studies that have manipulated growth conditions have shown that non-structural carbohydrate (NSC) reserves are restored rapidly after a disturbance, at the expense of growth activity^57,58^. This supports the empirical evidence that carbon storage takes priority over growth^59^, and trees maintain a safety margin in terms of carbohydrate reserves to cope with injuries caused by biotic or climatic events, including frost^60^. Moreover, shoot increment is primarily determined by the growth units already formed within the bud, which are influenced by the environmental conditions occurring during the preceding summer^61^. Considering that (1) frost damage negatively impacts individual resource acquisition^28,32,33^, (2) canopy recovery and restoration of NSC reserves have priority following a frost event, and (3) height growth is largely predetermined by the previous year’s environmental conditions, it is more likely to observe a decline in height growth in the year following the frost event and in individuals with a higher degree of damages, as highlighted in our study.

The damage caused by the late frost did not induce a phenological adjustment in our trees. Indeed, the timing of budbreak for the subsequent year remained similar between individuals of the same provenance, regardless of the frost damage levels. Bud phenology in black spruce provenances represents a local adaptation to the climatic conditions at the origin sites. Provenances from northern and colder sites exhibit an earlier budbreak than provenances from southern and warmer sites^37,62^, due to lower forcing requirements^50^. For this reason, our results indicate a maladaptive phenological plasticity and, even after a frost event, highly damaged trees do not adjust their phenology. In this context, provenances or individuals that are more susceptible to late frost damage also remain prone to suffer from similar events in the future.

Our results differ from observations of other non-lethal stresses, which resulted in phenological shifts in the following year^34–36^. This difference may be due to the degree of damage, which may not have been sufficient to alter sugar mobilization in the subsequent year. The limited number of buds produced in our damaged trees may not have created enough competition for sugars^63^. Although the trees may compensate for the damage at the expense of radial growth in the same year and height growth in the subsequent year, a prolonged carry-over effect on growth performance seems, to our knowledge, unlikely.

### A blessing in disguise or a curse in hiding?

Our results indicate that the functional traits studied in black spruce are related to the provenances, and thus probably affected by a local adaptation to the origin sites. When considering the phenological traits and the associated risk of late-spring frost exposure, northern provenances may be maladapted to future climatic conditions. However, assuming a certain degree of genetic variation within natural populations, this maladaptation could potentially serve as an evolutionary force that promotes a rapid local adaptation to the new environment. Given the magnitude of the ongoing climate change, adaptation would need to occur swiftly, which is a challenge for long-living organisms. Our results suggest that northern populations may have a faster generation turnover, which should foster a quicker evolutionary response to selective pressures compared with southern populations. A deeper assessment of reproductive traits such as seed production and germination rate is necessary to validate our hypothesis.

Similar considerations arise when contemplating the potential of moving species or provenances. The timing of first reproduction and generation turnover are vital traits to take into account in these practices. While further research is needed to evaluate variations in reproductive traits, migrating southern provenances to northern regions could potentially disrupt the long-standing adaptations to cope with recurring disturbances, such as wildfires. According to our results, southern provenances must reach an older age or larger size to reach reproductive maturity, which could impact the resilience of certain species in locations with more frequent and intense wildfires, like the northern latitudes of Quebec. So far, reproduction has received much less attention compared to growth-related traits, although it plays a critical role in ensuring the persistence of trees in specific locations and, consequently, the long-term success of the practices of forest management in a context of climate change.

## Materials and methods

### Provenances and common garden

The spruce provenances originated from five natural stands located along a latitudinal gradient that ranges from the 48th to 53rd parallels in the boreal coniferous forest of Quebec, Canada (Fig. 1). These provenances are Simoncouche (abbreviated as SIM), Bernatchez (BER), Mistassibi (MIS), Camp Daniel (DAN), and Mirage (MIR). The site origins represent natural black spruce stands lacking in anthropogenic disturbances or forest cuttings. The climate of the area is typically boreal, with long, cold winters and short, cool and wet summers. Temperatures decrease with increasing latitude and elevation, with the stands located at higher latitudes being the coldest in winter and least warm in summer. The annual temperature at the provenance origins ranges from − 3.4 to 1.2 °C.

In June 2012, we conducted sampling by randomly selecting 5–10 dominant trees in each stand and collecting 2–10 cones per tree, based on availability and accessibility of the canopy. On average, we collected 75–145 cones from each stand. In July 2014, we established the common garden by planting a total of 371 seedlings in a forest gap of 0.5 ha in SIM (48° 21′ 29″ N; 71° 23′ 59″ W) subjected to a clear-cut. We randomly planted the seedlings at a distance of 2 m × 2 m. We planted two rows of non-experimental spruces on each side of the plantation to avoid edge effects. The soil in the common garden is classified as podzolic, with a superficial organic layer reaching a depth of 10 cm. Based on the meteorological data collected from the weather station located at 500 m from the plantation, the average mean temperature during the study period (2015–2022) ranged from 1.55 to 3.29 °C. The year 2019 and 2021 were the coldest and the warmest years, respectively (Fig. S4). Throughout the study period, the number of months with a mean daily minimum temperature < 0 °C ranged from 6 to 8, with 2021 presenting freezing temperatures also in June (Fig. S4). Precipitation, in the form of rain, occurs mostly between late May and early October (Fig. S4).

For further details regarding the selection of provenances and the experimental design in the common garden see Silvestro et al.^37^.

### Data collection

We recorded bud break and bud set on a weekly basis from the beginning of May to the end of October during 2017–2022. To distinguish between the different phenological phases of the apical bud, we followed the procedure described by Dhont et al.^64^. We recorded (1) the onset of bud break, indicated by an open bud with a pale spot at the bud tip; (2) the end of bud break, indicated by an exposed shoot with needles fully emerged from the surrounding scales and spreading outwards; (3) the onset of bud set, indicated by the presence of a white bud; and (4) the end of bud set, indicated by a fully formed bud with the needles in the whorl spreading outwards. Shoot extension corresponds to the period of annual growth of the apical arrow, occurring between the end of bud burst and the onset of bud set^24^.

At the end of the growing season 2022, we recorded the total height and stem diameter at the collar of each tree. To track annual growth of the primary meristem, we measured shoot extension during 2017–2022 on the internodes of the main stem using a measuring tape, with a precision of 2 mm. We also identified the trees reaching reproductive maturity, based on the presence of cones, from 2020 (the first year in which cones were observed on the trees) to 2022.

### Late frost damage assessment

We conducted a visual assessment of damage after a late frost occurring in June 2021 using the protocol on browning for conifers^65^. We estimated the proportion of damaged brown buds out of the total buds on each tree and defined three damage levels based on the classes: (0) no damaged bud; (1) low, < 15% of buds damaged; (2) high, > 15% of buds damaged. Additional details regarding the late frost in 2021, the assessment of frost damage at the study site, and the amount of damaged trees are available in Mura et al.^50^.

### Statistical analyses

We evaluated the effect of annual temperature at the provenance site on the timings of each phenological phase and their durations by performing an Analysis of Covariance (ANCOVA) using Ordinary Least Squares (OLS) regressions. We compared tree height and diameter among provenances using a one-way analysis of variance (ANOVA), and Tukey’s HSD tests for multiple comparisons. We assessed the relationships between reproductive stage and both total height and basal diameter using a two-way ANOVA, including the origin site and reproductive stage as categorical variables.

We investigated the effect of frost damage on bud phenology and shoot growth with a two-way ANOVA. Origin site and frost damage level were included as categorical variables.

We quantified the impact of high-intensity frost damage on growth performance and bud phenology on the two provenances showing the highest level of damage (i.e., > 15% of damaged buds), DAN and MIR. For each of these two provenances, we compared growth and phenology between damaged and undamaged trees during 2017–2022 by using a two-way ANOVA. To assess the effect of frost on the timings of bud phenology, we performed a two-way ANOVA for each phenological stage. Statistics were performed in R version 4.2.2^66^.

### Supplementary Information

Supplementary Information.

## Data Availability

Data associated with this paper are available in Borealis: 10.5683/SP3/EYBESE.

## References

[1] Stott P (2016). How climate change affects extreme weather events. Science.

[2] Reichstein M (2013). Climate extremes and the carbon cycle. Nature.

[3] IPCC. Climate Change 2022: Impacts, Adaptation and Vulnerability (Cambridge University Press, Cambridge, UK and New York, NY, USA, 2022).

[4] McKenney DW, Pedlar JH, Rood RB, Price D (2011). Revisiting projected shifts in the climate envelopes of North American trees using updated general circulation models. Glob. Change Biol..

[5] Sittaro F, Paquette A, Messier C, Nock CA (2017). Tree range expansion in eastern North America fails to keep pace with climate warming at northern range limits. Glob. Change Biol..

[6] Boshier D (2015). Is local best? Examining the evidence for local adaptation in trees and its scale. Environ. Evid..

[7] Park A, Talbot C (2018). Information underload: Ecological complexity, incomplete knowledge, and data deficits create challenges for the assisted migration of forest trees. BioScience.

[8] Maynard DS (2022). Global relationships in tree functional traits. Nat. Commun..

[9] O'Brien EK, Mazanec RA, Krauss SL (2007). Provenance variation of ecologically important traits of forest trees: Implications for restoration. J. Appl. Ecol..

[10] Aubin I (2016). Traits to stay, traits to move: A review of functional traits to assess sensitivity and adaptive capacity of temperate and boreal trees to climate change. Environ. Rev..

[11] Savolainen O, Pyhäjärvi T, Knürr T (2007). Gene flow and local adaptation in trees. Annu. Rev. Ecol. Evol. Syst..

[12] Aitken SN, Yeaman S, Holliday JA, Wang T, Curtis-McLane S (2008). Adaptation, migration or extirpation: Climate change outcomes for tree populations. Evol. Appl..

[13] Pelletier E, De Lafontaine G (2023). Jack pine of all trades: Deciphering intraspecific variability of a key adaptive trait at the rear edge of a widespread fire-embracing North American conifer. Am. J. Bot..

[14] de Lafontaine G, Napier JD, Petit RJ, Hu FS (2018). Invoking adaptation to decipher the genetic legacy of past climate change. Ecology.

[15] Körner C, Basler D (2010). Phenology under global warming. Science.

[16] Chuine I (2010). Why does phenology drive species distribution?. Philos. Trans. R. Soc. B Biol. Sci..

[17] Wang Y (2019). Frost controls spring phenology of juvenile Smith fir along elevational gradients on the southeastern Tibetan Plateau. Int. J. Biometeorol..

[18] Savolainen, O. *et al.* in *EUFORGEN Climate Change and Forest Genetic Diversity: Implications for Sustainable Forest Management in Europe, Paris, France, 15–16 March 2006, 19–30.* (eds J. Koskela, A. Buck, & E. Teissier du Cros) 19–30 (Biodiversity International, Rome, Italy, 2007).

[19] Mlambo MC (2014). Not all traits are ‘functional’: Insights from taxonomy and biodiversity-ecosystem functioning research. Biodivers. Conserv..

[20] Parmesan C, Hanley ME (2015). Plants and climate change: Complexities and surprises. Ann. Bot..

[21] Harper J, White J (1974). The demography of plants. Annu. Rev. Ecol. Syst..

[22] Rice KJ, Emery NC (2003). Managing microevolution: Restoration in the face of global change. Front. Ecol. Environ..

[23] Owens JN (1995). Constraints to seed production: Temperate and tropical forest trees. Tree Physiol..

[24] Silvestro R, Brasseur S, Klisz M, Mencuccini M, Rossi S (2020). Bioclimatic distance and performance of apical shoot extension: Disentangling the role of growth rate and duration in ecotypic differentiation. For. Ecol. Manag..

[25] Frank A (2017). Distinct genecological patterns in seedlings of Norway spruce and silver fir from a mountainous landscape. Ecology.

[26] St Clair JB, Mandel NL, Vance-Borland KW (2005). Genecology of Douglas fir in Western Oregon and Washington. Ann. Bot..

[27] Montwé D, Isaac-Renton M, Hamann A, Spiecker H (2018). Cold adaptation recorded in tree rings highlights risks associated with climate change and assisted migration. Nat. Commun..

[28] Vitasse Y, Lenz A, Körner C (2014). The interaction between freezing tolerance and phenology in temperate deciduous trees. Front. Plant Sci..

[29] Charrier G, Bonhomme M, Lacointe A, Améglio T (2011). Are budburst dates, dormancy and cold acclimation in walnut trees (*Juglans regia* L.) under mainly genotypic or environmental control?. Int. J. Biometeorol..

[30] Liu Q (2018). Extension of the growing season increases vegetation exposure to frost. Nat. Commun..

[31] Marquis B, Bergeron Y, Houle D, Leduc M, Rossi S (2022). Variability in frost occurrence under climate change and consequent risk of damage to trees of western Quebec, Canada. Sci. Rep..

[32] Vitasse Y, Lenz A, Hoch G, Körner C (2014). Earlier leaf-out rather than difference in freezing resistance puts juvenile trees at greater risk of damage than adult trees. J. Ecol..

[33] Vitasse Y, Schneider L, Rixen C, Christen D, Rebetez M (2018). Increase in the risk of exposure of forest and fruit trees to spring frosts at higher elevations in Switzerland over the last four decades. Agric. For. Meteorol..

[34] Ren P (2020). Warming counteracts defoliation-induced mismatch by increasing herbivore-plant phenological synchrony. Glob. Change Biol..

[35] Deslauriers A, Fournier M-P, Cartenì F, Mackay J (2018). Phenological shifts in conifer species stressed by spruce budworm defoliation. Tree Physiol..

[36] Falk MA, Donaldson JR, Stevens MT, Raffa KF, Lindroth RL (2020). Phenological responses to prior-season defoliation and soil-nutrient availability vary among early- and late-flushing aspen (*Populus tremuloides* Michx.) genotypes. For. Ecol. Manag..

[37] Silvestro R (2019). From phenology to forest management: Ecotypes selection can avoid early or late frosts, but not both. For. Ecol. Manag..

[38] Stearns, S. C. in *Conceptual Change in Biology: Scientific and Philosophical Perspectives on Evolution and Development* (ed Alan C. Love) 131–146 (Springer Netherlands, 2015).

[39] Thomas, S., Meinzer, F., Lachenbruch, B. & Dawson, T. Size-and age-related changes in tree structure and function. in *Size-and Age-Related Changes in Tree Structure and Function *33–64 (Springer Science+ Business Media BV, Dordrecht, 2011).

[40] Andrus RA, Harvey BJ, Hoffman A, Veblen TT (2020). Reproductive maturity and cone abundance vary with tree size and stand basal area for two widely distributed conifers. Ecosphere.

[41] Viglas JN, Brown CD, Johnstone JF (2013). Age and size effects on seed productivity of northern black spruce. Can. J. For. Res..

[42] Davi H (2016). Disentangling the factors driving tree reproduction. Ecosphere.

[43] Santos-Del-Blanco L, Climent J, González-Martínez SC, Pannell JR (2012). Genetic differentiation for size at first reproduction through male versus female functions in the widespread Mediterranean tree *Pinus pinaster*. Ann. Bot..

[44] Kozłowski J (1992). Optimal allocation of resources to growth and reproduction: Implications for age and size at maturity. Trends Ecol. Evol..

[45] Alfaro-Sánchez R (2022). What drives reproductive maturity and efficiency in serotinous boreal conifers?. Front. Ecol. Evol..

[46] Johnstone JF, Chapin FS (2006). Fire interval effects on successional trajectory in boreal forests of northwest Canada. Ecosystems.

[47] Dawe DA, Parisien M-A, Van Dongen A, Whitman E (2022). Initial succession after wildfire in dry boreal forests of northwestern North America. Plant Ecol..

[48] Pausas JG, Keeley JE (2014). Evolutionary ecology of resprouting and seeding in fire-prone ecosystems. New Phytologist.

[49] Oris F (2014). Long-term fire history in northern Quebec: Implications for the northern limit of commercial forests. J. Appl. Ecol..

[50] Mura C (2022). The early bud gets the cold: Diverging spring phenology drives exposure to late frost in a *Picea mariana* [(Mill.) BSP ] common garden. Physiol. Plant..

[51] Rubio-Cuadrado Á (2021). Impact of successive spring frosts on leaf phenology and radial growth in three deciduous tree species with contrasting climate requirements in central Spain. Tree Physiol..

[52] Vitasse Y (2019). Contrasting resistance and resilience to extreme drought and late spring frost in five major European tree species. Glob. Change Biol..

[53] Príncipe A (2017). Low resistance but high resilience in growth of a major deciduous forest tree (*Fagus sylvatica* L.) in response to late spring frost in southern Germany. Trees.

[54] Baumgarten F, Gessler A, Vitasse Y (2023). No risk—No fun: Penalty and recovery from spring frost damage in deciduous temperate trees. Funct. Ecol..

[55] D'Andrea E (2019). Winter's bite: Beech trees survive complete defoliation due to spring late-frost damage by mobilizing old C reserves. New Phytol..

[56] Vander Mijnsbrugge K (2021). Growth recovery and phenological responses of juvenile beech (*Fagus sylvatica* L.) exposed to spring warming and late spring frost. Forests.

[57] Weber R (2018). Living on next to nothing: Tree seedlings can survive weeks with very low carbohydrate concentrations. New Phytol..

[58] Schönbeck L (2018). Homeostatic levels of nonstructural carbohydrates after 13 yr of drought and irrigation in *Pinus sylvestris*. New Phytol..

[59] Sala A, Woodruff DR, Meinzer FC (2012). Carbon dynamics in trees: Feast or famine?. Tree Physiol..

[60] Klein T, Vitasse Y, Hoch G (2016). Coordination between growth, phenology and carbon storage in three coexisting deciduous tree species in a temperate forest. Tree Physiol..

[61] Salminen H, Jalkanen R (2005). Modelling the effect of temperature on height increment of Scots pine at high latitudes. Silva Fennica.

[62] Guo X (2021). Common-garden experiment reveals clinal trends of bud phenology in black spruce populations from a latitudinal gradient in the boreal forest. J. Ecol..

[63] Barbier FF, Lunn JE, Beveridge CA (2015). Ready, steady, go! A sugar hit starts the race to shoot branching. Curr. Opin. Plant Biol..

[64] Dhont, P., Sylvestre, P., Gros-Louis, M. C. & Isabel, N. Field Guide for Identifying Apical Bud Break and Bud Formation Stages in White Spruce (2010).

[65] Burr, K. E. *et al.* in *Conifer Cold Hardiness Tree Physiology* Ch. Chapter 14, 369–401 (Springer, Dordrecht, 2001).

[66] R: A language and environment for statistical. R Foundation for Statistical Computing, Vienna, Austria (2022).

