# Supplementary material for: Local adaptation shapes functional traits and resource allocation in black spruce
Source: Sci Rep. 2023 Dec 1;13:21257. doi: 10.1038/s41598-023-48530-6 (PMC10692160; doi:10.1038/s41598-023-48530-6)
Supplement: Supplementary file 1 — Supplementary Information. [file 41598_2023_48530_MOESM1_ESM.docx]

**Annexes**

**Title:**

Local adaptation shapes functional traits and resource allocation in black spruce.

**Authors**

Silvestro, R.^1, *^, Mura, C. ^1^, Alano Bonacini, D. ^1^, de Lafontaine, G.^2^, Faubert, P. ^1,3^, Mencuccini, M.^4,5^, Rossi, S^1^.

**Affiliations**

^1^ Laboratoire sur les écosystèmes terrestres boréaux, Département des Sciences Fondamentales, Université du Québec à Chicoutimi, 555 boulevard de l’Université, Chicoutimi, QC G7H2B1, Canada.

^2^ Canada Research Chair in Integrative Biology of the Northern Flora, Département de biologie, chimie et géographie, Centre for Northern Studies, Centre for Forest Research, Université du Québec à Rimouski, Rimouski, Québec, Canada.

^3^ Carbone boréal, Département des Sciences Fondamentales, Université du Québec à Chicoutimi, 555 boulevard de l’Université, Chicoutimi, QC G7H 2B1, Canada.

^4^ Centre de Recerca Ecològica i Aplicacions Forestals (CREAF), 08193 Bellaterra, Barcelona, Spain.

^5^ Institució Catalana de Recerca i Estudis Avançats (ICREA), Passeig de Lluis Companys 23, 08010 Barcelona, Spain.

^*^ Corresponding author: [roberto.silvestro1@uqac.ca](mailto:roberto.silvestro1@uqac.ca) (RS)

**Table S1** Effects of mean annual temperature of the provenance (Temp), Year and their interaction (Temp×Year) on the phenological phases evaluated by ANCOVA models.

|  | Phase | R^2^ |  | Effects | | |
| --- | --- | --- | --- | --- | --- | --- |
|  |  |  |  | Temp | Year | Temp x Year |
| **Bud break** | Onset | 0.63 |  | 302.70*** | 978.28*** | 2.45** |
|  | Ending | 0.51 |  | 492.35*** | 1182.11*** | 3.27* |
|  | Duration | 0.19 |  | 28.95*** | 342.17*** | 10.01* |
|  |  |  |  |  |  |  |
| **Growing season** | Duration | 0.14 |  | 29.31*** | 136.82*** | 16.88** |
|  |  |  |  |  |  |  |
| **Bud set** | Onset | 0.73 |  | 116.41*** | 954.77*** | 9.92** |
|  | Ending | 0.20 |  | 97.04*** | 276.88*** | 13.67* |
|  | Duration | 0.30 |  | 30.89*** | 546.35*** | 4.52* |

* *P* < 0.05; ** *P* < 0.01; *** *P* < 0.001.

| **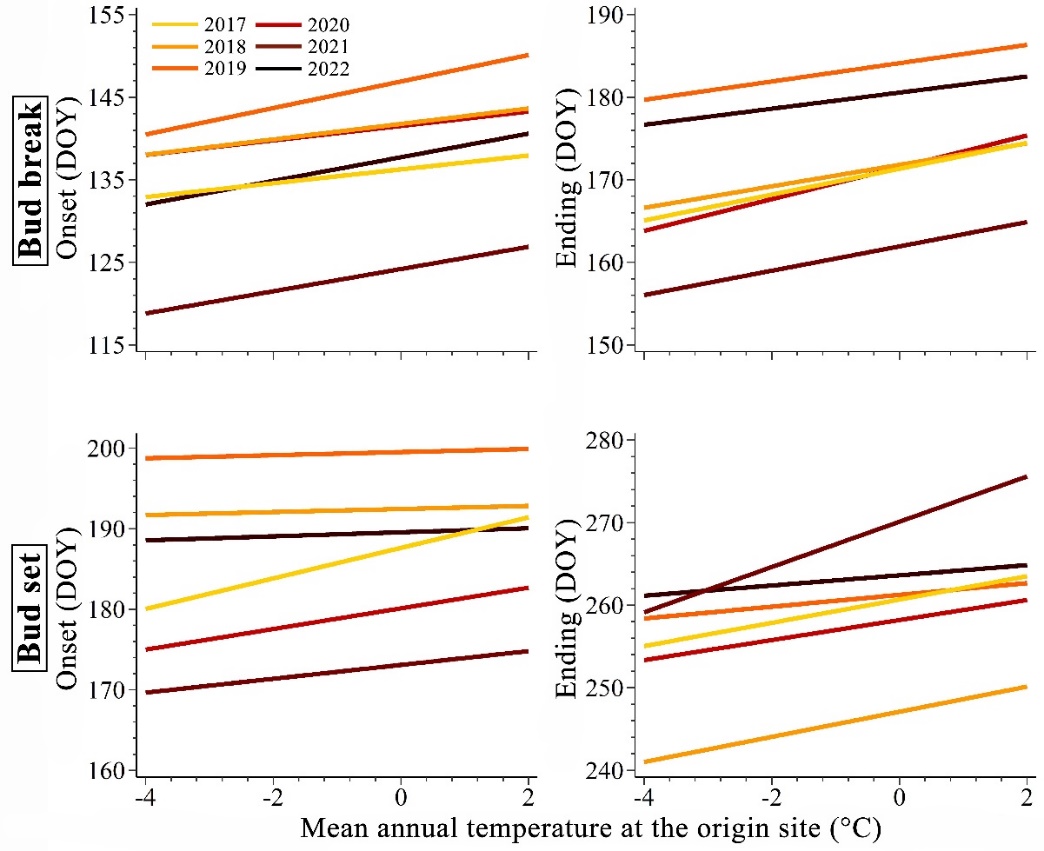** |
| --- |

**Figure S1** Days of occurrence of the onset and the ending for both bud burst and bud set for each black spruce provenance predicted by the Ordinary Least Squares (OLS) models.

| **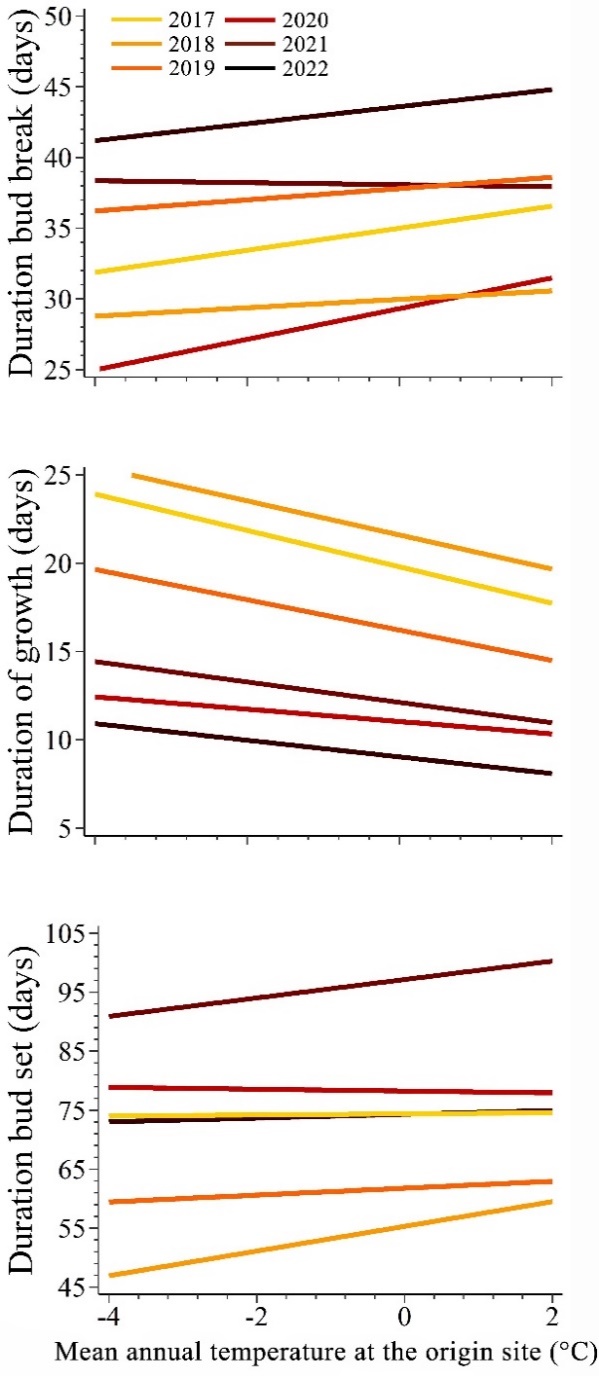** |
| --- |

**Figure S2** Duration of bud burst, shoot extension, and bud set for each black spruce provenance predicted by the Ordinary Least Squares (OLS) models.

| **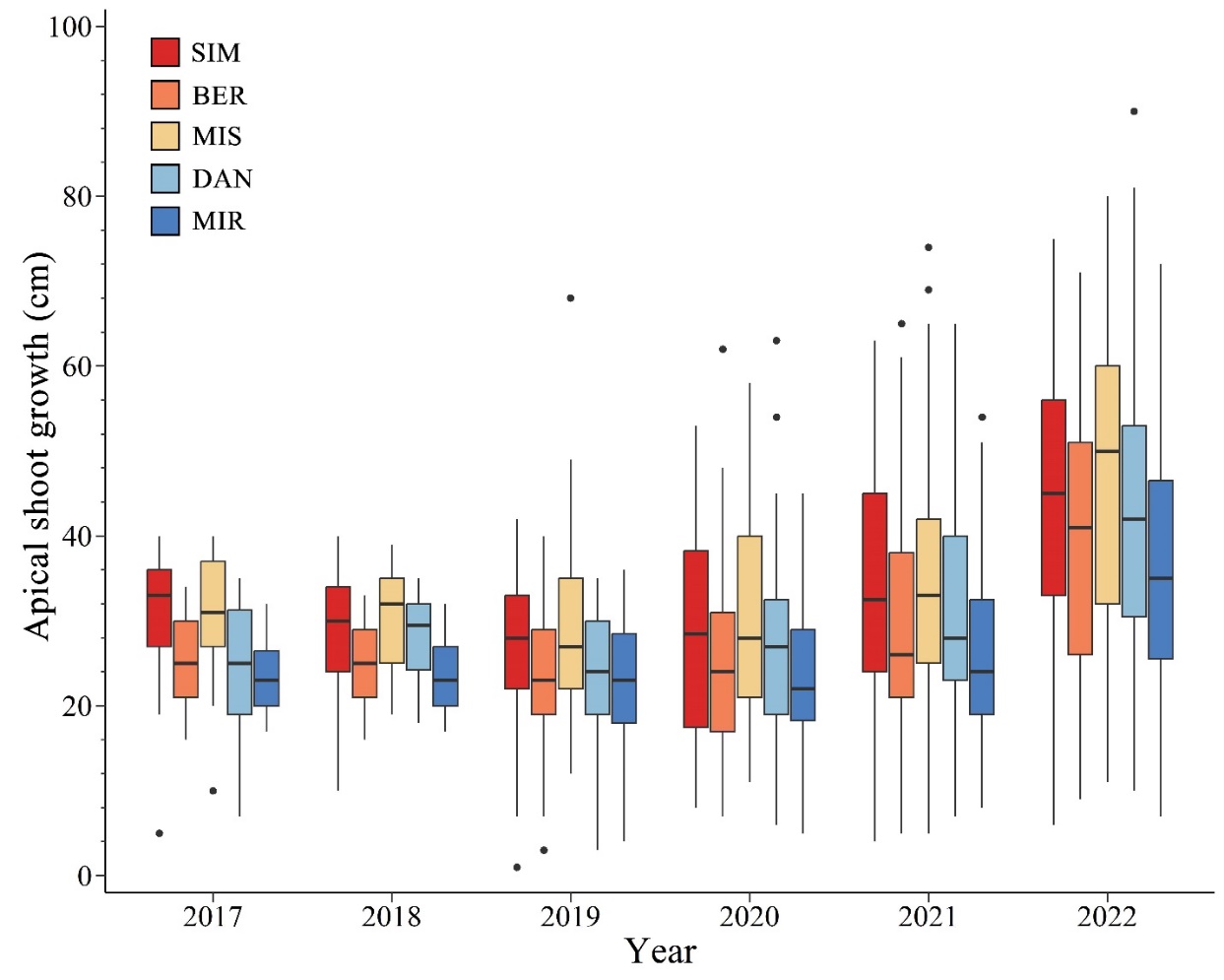** |
| --- |

**Figure S3** Apical shoot growth for each black spruce provenance during the study years 2017-2022.

|  | R^2^ |  | Effects | | |
| --- | --- | --- | --- | --- | --- |
|  |  |  | Prov | Repr | Prov x Repr |
| **Diameter** | 0.17 |  | 6.77*** | 35.68*** | 2.96* |
| **Height** | 0.19 |  | 6.11*** | 56.07*** | 3.74* |

**Table S2** Effects of the provenance (Prov), reproductive maturity (Repr) and their interaction (Prov × Repr) on basal diameter and total height evaluated by ANCOVA models.

* *P* < 0.05; ** *P* < 0.01; *** *P* < 0.001.

**Table S3** Effects of the Year, damage level (Damage) and their interaction (Year x Damage) on the timing of onset and ending for both bud break and bud set evaluated by ANOVA models.

|  |  | Phase | R^2^ |  | Effects | | |
| --- | --- | --- | --- | --- | --- | --- | --- |
|  |  |  |  |  | Year | Damage | Year x Damage |
| **MIR** | **Bud break** | Onset | 0.70 |  | 246.34*** | 0.96 | 1.18 |
|  |  | Ending | 0.53 |  | 312.93*** | 0.01 | 0.19 |
|  |  |  |  |  |  |  |  |
|  | **Bud set** | Onset | 0.80 |  | 337.50*** | 2.73 | 0.68 |
|  |  | Ending | 0.15 |  | 65.57*** | 1.58 | 1.58 |
|  |  |  |  |  |  |  |  |
| **DAN** | **Bud break** | Onset | 0.63 |  | 192.76*** | 7.22 | 0.83 |
|  |  | Ending | 0.52 |  | 256.75*** | 0.04 | 3.18 |
|  |  |  |  |  |  |  |  |
|  | **Bud set** | Onset | 0.85 |  | 352.58*** | 5.73 | 9.75 |
|  |  | Ending | 0.11 |  | 44.67*** | 0.14 | 1.17 |

* *P* < 0.05; ** *P* < 0.01; *** *P* < 0.001.

**Table S4** Effects of the provenance (Prov), damage level (Damage) and their interaction (Prov x Damage) on growth performances evaluated by ANOVA model.

| R^2^ |  | Effects | | |
| --- | --- | --- | --- | --- |
|  |  | Prov | Damage | Prov x Damage |
| 0.21 |  | 4.73** | 5.85** | 2.01** |

* *P* < 0.05; ** *P* < 0.01; *** *P* < 0.001.

**Table S5** Effects of the year, damage level (Damage) and their interaction (Year x Damage) on growth performances for Mirage (MIR) and Camp Daniel (DAN) provenances evaluated by ANOVA models.

| Provenance | Phase | R^2^ |  | Effects | | |
| --- | --- | --- | --- | --- | --- | --- |
|  |  |  |  | Year | Damage | Year x Damage |
| **MIR** | Onset | 0.26 |  | 18.99*** | 0.01 | 4.80*** |
| **DAN** | Ending | 0.30 |  | 28.32*** | 0.75 | 3.72** |

* *P* < 0.05; ** *P* < 0.01; *** *P* < 0.001.

| 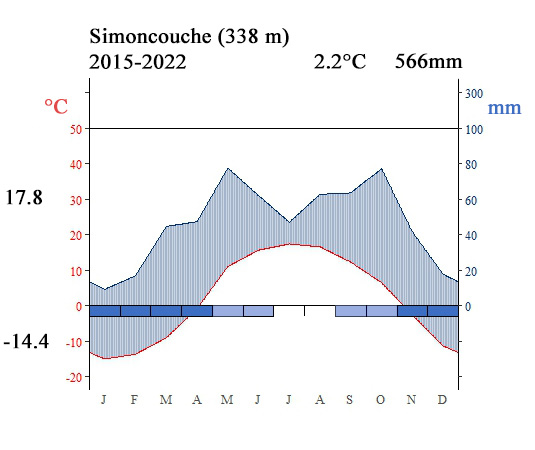 |
| --- |

**Figure S4** Walter and Lieth climatic diagram for the common garden.
